# Well-Differentiated Liposarcoma That Increased in Size after Menopause: A Case Report and a Review of the Literature

**DOI:** 10.1155/2024/7599714

**Published:** 2024-02-24

**Authors:** Tatsuji Hoshino, Yoshihiro Takada, Ayako Sugihara, Masato Kinugasa, Yoshiyuki Tsuji

**Affiliations:** ^1^Department of Obstetrics and Gynecology, Meiwa General Hospital, Nishinomiya, Japan; ^2^Department of Radiology, Meiwa General Hospital, Nishinomiya, Japan; ^3^Department of Pathology, Meiwa General Hospital, Nishinomiya, Japan

## Abstract

This study reports a case of uterine liposarcoma together with a literature review. At 52 years old, our patient was diagnosed with lipoleiomyoma by MRI. A mass (39 × 32 × 41 mm^3^) protruding from the anterior wall of the uterine body was observed. When the patient was 58, her previous doctor found that the tumor had grown, and she was referred to the gynecology department of our hospital. On MRI, the major diameter was 1.23-fold longer and the volume was 1.85-fold higher compared with the prior imaging findings. Diffusion-weighted images revealed no significant anomalous signals. Thus, malignant tumors were included in the differential diagnosis. The patient consented to total abdominal hysterectomy and bilateral salpingo-oophorectomy. The mass on the anterior wall remained completely in the myometrium. No implantation was found in the abdominal cavity, and ascites was not detected. No bleeding or necrosis was observed on the cut surface. Histopathologically, differences in the sizes of adipocytes and stromal cells were identified. There were irregularities in the nuclear findings. The immunohistochemical findings were as follows: CDK4 (+), desmin (+), S100p (−), and Ki − 67 = 1%. Therefore, a diagnosis of well-differentiated liposarcoma was rendered. The lesion was localized in the uterus, and it was completely removed during surgery. Well-differentiated liposarcoma of uterine primary has no possibility of recurrence following complete resection, and thus, the patient underwent follow-up without additional treatment. No metastasis or recurrence has been observed for 10 months after surgery.

## 1. Introduction

The differential diagnosis of lipoleiomyoma and liposarcoma of the uterus is difficult. On imaging, some of the masses that characterize uterine lipoleiomyoma can exhibit a developmental form with reduced diffusion on diffusion-weighted images. Such lesions infiltrate the muscle layer and progress outside the uterus on MRI. In such cases, the possibility of liposarcoma might be considered, but the definitive diagnosis depends on the results of histopathological examination. In the present case, lipoleiomyoma was suspected before surgery and follow-up, but after postmenopausal observation, the lesion increased in size. After surgical removal, the lesion was histopathologically diagnosed as liposarcoma. The case has been reported with a literature review [1–16].

## 2. Case Presentation

The woman in the case (G4P2) experienced menopause at 49 years old. At the age of 52, she visited her doctor, and she was referred to our hospital because of an ovarian cyst. Transvaginal ultrasound (TVUS) revealed fibroids with a major diameter of 40 mm, and MRI was performed to distinguish ovarian tumors and fibroids.

**Figure 1:**
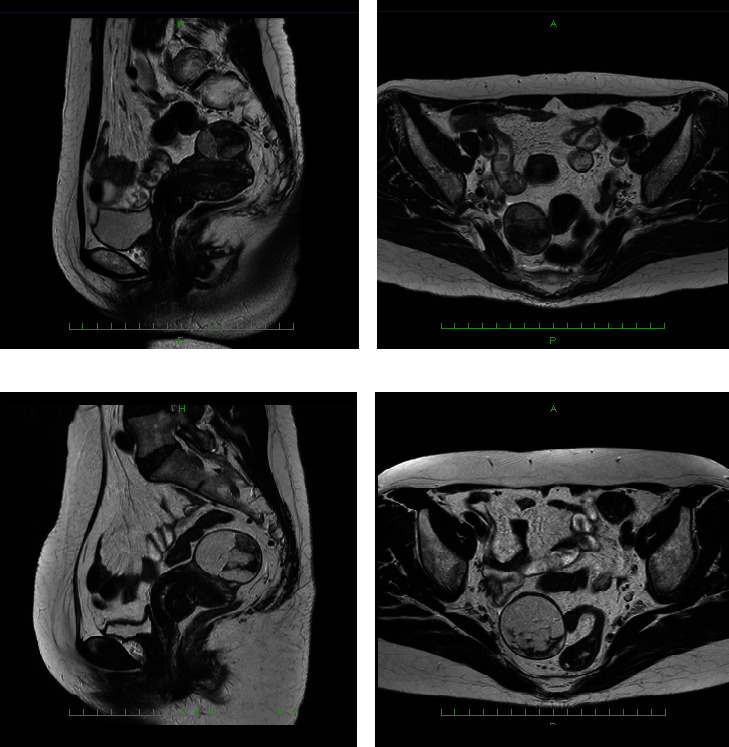
Preoperative MRI at 52 and 58 years old: (a) T2-weighted image at 52 years old presenting the maximum section of the mass in sagittal plane; (b) T2-weighted image presenting the maximum section of the mass in the transverse plane; (c) T2-weighted image at 58 years old presenting the maximum section of the mass in the sagittal plane; (d) T2-weighted image presenting the maximum section of the mass in the transverse plane. The average diameters of the major and minor axes on the sagittal plane and the major diameter of the transverse plane increased by 1.23-fold. The tumor volume increased from 27 to 49 mL (1.85-fold) when approximated by the elliptical sphere formula.

On MRI at 52 (Figures 1(a) and 1(b)), a mass measuring 39 × 32 × 41 mm^3^ protruded from the anterior wall of the uterine body. The internal signal was uneven, and both the T1- and T2-weighted images had a mixture of low-intensity areas with faint high-intensity areas. The pale and hyperintense area had a suppressed signal on fat suppression images, and it was considered a fat component. The subseries fibroid of the anterior wall of the uterine body was considered lipoleiomyoma. In addition, fibroids were noted.

After MRI, we explained the possibility of lipoleiomyoma to the patient, and because she was menopausal, we decided to perform a cytological examination and TVUS for fibroids every year. Thereafter, gynecological examination, cervical cytology, and TVUS were performed annually. However, cytology was negative for intraepithelial lesion or malignancy, and no significant changes were observed in the gynecological examination or TVUS findings. At the age of 58, the patient's previous doctor detected an increase in the size of the lesion, and she was referred to the gynecology department of our hospital to determine whether the lesion was a uterine fibroid or ovarian tumor. Because clear enlargement was detected, MRI was performed.

**Figure 2:**
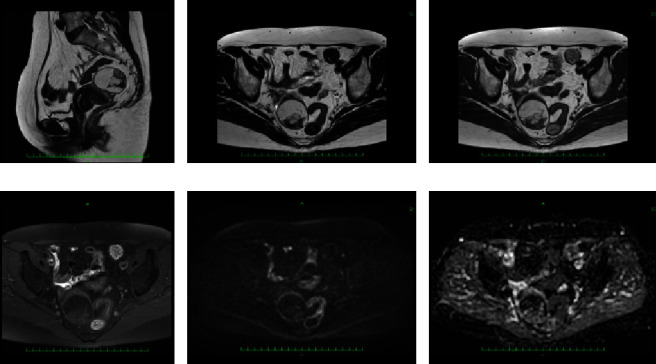
MRI at 58 years old: (a) T2-weighted image of the sagittal plane; (b) T2-weighted image of the transverse plane; (c) T1-weighted image of the transverse plane; (d) fat-suppressed T1-weighted image of the transverse plane; (e) diffusion-weighted image of the transverse plane; (f) apparent diffusion coefficient image of the transverse plane. The mass of the anterior uterine wall that was highly intense on T1- and T2-weighted images was considered a fatty component because the signal was suppressed on fat-suppressed imaging. Diffusion-weighted imaging revealed no obvious abnormal findings, suggesting that the cell density of the mass was low and was unlikely to be a malignant finding.

The findings of MRI when the patient was 58 (Figures 1(c) and 1(d) and 2) included retroflection of the uterus. A tumor lesion measuring 49 × 40 × 49 mm^3^ protruded into the anterior wall of the uterus. The internal signal was uneven, with high- and low-intensity areas observed on T1- and T2-weighted images. The majority of masses were hyperintense on T1- and T2-weighted imaging, and signal suppression was observed on fat-suppressed T1-weighted imaging. Compared with the results of MRI performed when the patient was 52, the major diameter was 1.23-fold longer, and the volume was 1.85-fold higher (from 27 mL to 49 mL). Diffusion-weighted images revealed no significant anomalous signals. The lesion was considered a subserous lipoleiomyoma. In addition, fibroids were observed in the muscle layer.

Although the patient was menopausal, the tumor significantly increased in size, and thus, we explained the possibility of malignancy to the patient and her family. The patient and her family consented to total abdominal hysterectomy and bilateral salpingo-oophorectomy (TAH/BSO). Preoperative examinations revealed no significant changes. The patient's lactate dehydrogenase and hemoglobin levels were 165 U/L and 13.8 g/dL, respectively.

**Figure 3:**
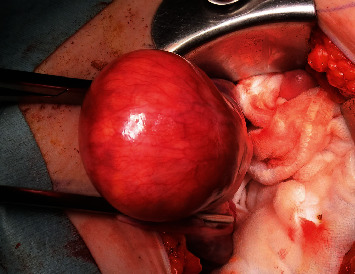
Operative findings during laparotomy. The photograph was taken from the left side of the patient. The left side is the caudal side, the right side is the cephalic side, the front side is the left side, and the opposite side is the right side. A mass localized near the bottom of the uterus was noted. Yellow masses were seen through the thinned myometrium. No rupture was observed in the myometrium, no dissemination foci were found in the abdominal cavity, and ascites was not detected.

Under general anesthesia, a transverse incision of approximately 14 cm was made above the pubic bone, and laparotomy was performed as usual. TAH/BSO was subsequently performed. The weight of the extracted lesion was 128 g, and the mass on the anterior wall remained completely in the myometrium (Figure 3). When the anterior wall of the uterus was incised, the mass was soft and yellow in color. No bleeding or necrosis was observed on the cut surface. No implantation or adhesions were found in the abdominal cavity, and ascites was not observed.

**Figure 4:**
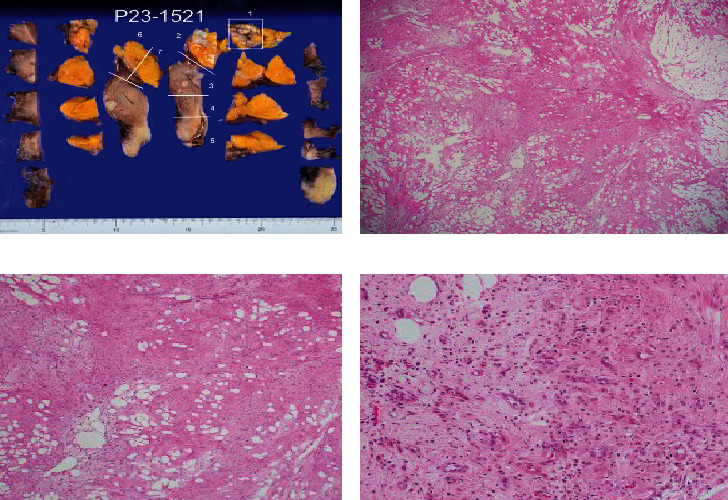
Macroscopic findings and hematoxylin–eosin (HE) staining findings: (a) display of the pathological specimen site after ALTFiX fixation; (b) HE staining of specimen preparation site 1 (×100); (c) HE staining of specimen preparation site 1 (×200); (d) HE staining of specimen preparation site 1 (×400). Differences in the sizes of adipocytes and stromal cells were noted (b, c). There were irregularities in the nuclear findings of stromal cells and irregularities and thickening of the nuclear margins. Nuclear enrichment was observed (d).

**Figure 5:**
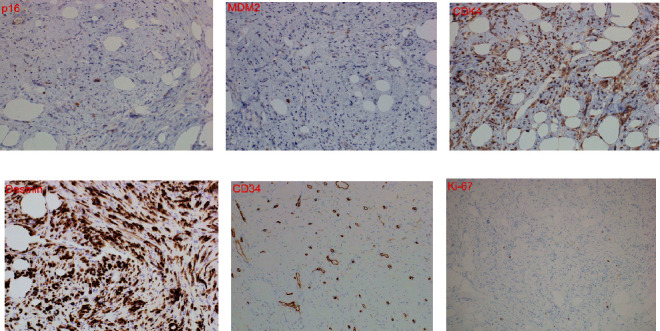
Immunohistochemical staining: (a) p16 (×400). Some cytoplasm and nuclei were stained; (b) MDM2 (×400). A small part of the cytoplasm and nucleus was stained; (c) CDK4 (×400). The cytoplasm of long spindle-shaped cells of the connective tissue and the cytoplasm of adipocytes were stained; (d) desmin (×400). The cytoplasm of long spindle-shaped cells of the connective tissue and the cytoplasm of adipocytes were stained; (e) CD34 (×400). Some cell membranes were stained; (f) Ki-67 index = 1%.

A continuous yellow tumor of 50 × 40 mm^2^ in size was detected in the uterine body. On the split surface, a partially white tone induration was noted. Histologically, short spindle-shaped tumor cells grew in coarse bundles in fibroadipose tissue (Figure 4). Differences in the sizes of adipocytes and stromal cells were noted (Figures 4(b) and 4(c)). There were irregularities in the nuclear findings of the stromal cells, including thickening of the nuclear margins. Nuclear enrichment was also observed (Figure 4(d)). The immunohistochemical findings were as follows (Figure 5): p16 (partially +), MDM2 (only a small part +), CDK4 (+), desmin (+), S100p (−), CD34 (partially +), and Ki-67 index of 1%. Based on these findings, the lesion was diagnosed as well-differentiated liposarcoma. Multiple fibroids were observed in the muscle layer. No obvious malignant findings were found in the endometrium, cervix, or bilateral adnexa. The pathological diagnosis was well-differentiated liposarcoma and multiple leiomyomas.

The lesion was localized in the uterus and was completely removed. From the previous reports, well-differentiated liposarcoma with a primary retroperitoneal location is prone to local recurrence because of the difficulty in complete resection, but well-differentiated liposarcoma with a primary uterine location does not recur if it is completely resected. Therefore, the patient underwent follow-up without additional treatment. Postoperative PET-CT revealed no evidence of metastasis or recurrence (data not shown). Recurrence and metastasis have not been observed over 10 months after surgery.

## 3. Discussion

Lipomas, lipoleiomyomas, and liposarcomas are similar in appearance. Lipomas have an appearance similar to adipose tissue, whereas liposarcomas are somewhat grayish white and mucus-like. Lipomas consist of microscopically mature adipose tissue, and they cannot be distinguished from normal adipose tissue. Tumor cells consist of small mature fat cells that cannot be distinguished from normal fat cells. In liposarcoma, tumor cells grow in a connective weave containing a network of intricately branching delicate capillaries. Nuclear atypia can be observed, and tumor cells with densely stained atypical nuclei are scattered [17]. Various sarcomas develop in the uterus, albeit less frequently than carcinoma. The two major uterine sarcomas are leiomyosarcoma and endometrial stromal sarcoma. Others include Kaposi sarcoma, rhabdomyosarcoma, angiosarcoma, and liposarcoma, although they are rare tumors [18].

According to the WHO Classification of Tumors, 5th edition, liposarcoma is divided into five histological types: well-differentiated, dedifferentiated, myxoid, round cell, and polymorphic. Well-differentiated liposarcoma is an intermediate group (locally invasive) tumor that exhibits adipocyte differentiation. Well-differentiated liposarcoma most commonly occurs in middle-aged and elderly women. It arises preferentially in the deep soft tissues of the extremities, especially the thighs, followed by the retroperitoneal and paratesticular regions. It can also occur in the mediastinum and in subcutaneous regions. In the extremities, well-differentiated liposarcoma manifests as a painless mass that gradually increases in size over several months to years. In the retroperitoneum, well-differentiated liposarcoma presents as a symptomatic intraperitoneal mass, or it is discovered by chance. In the retroperitoneum, tumor diameters often exceed 20 cm. Complete resection is more feasible for lesions located in the extremities than for those located in the retroperitoneum, and thus, the local recurrence rate for the former tumors is low. Conversely, almost all patients with retroperitoneal disease experience recurrence. Repeated recurrence increases the risk of dedifferentiation. Dedifferentiation occurs in more than 20% of retroperitoneal lesions, versus no more than 5% of lesions in the extremities. Over a long period, more than 80% of patients with retroperitoneal liposarcoma die of the disease, versus 0% of patients with liposarcoma of the extremities [17]. In retroperitoneal and limb liposarcoma, the myxoid and well-differentiated types are classified as relatively low-grade tumors because they have a strong tendency to recur locally but rarely cause distant metastasis. Contrarily, the round cell and polymorphic types cause distant metastasis early, and thus, they are categorized into a high-grade with a poor prognosis [17, 18].

On MRI, findings indicative of benign lipoma include homogeneous adipose tissue and a small number of thin distinct septa. The MRI findings of well-differentiated liposarcoma include a thick nodular septum of 2 mm or more, nonfatty areas, prominent hyperintense areas on T2-weighted imaging, and prominent areas on contrast enhancement [15, 19, 20]. In this case, when the MR images were reviewed again after knowing the results, it was noticed that the septum, which is the binding weave component between the fat components, was somewhat thickened and irregular (Figures 1, 2, and 4(a)). This finding could be suspicious for liposarcoma.

In this case, the major axis diameter increased by 1.23-fold over the course of 6 years, and the volume increased from 27 to 49 mL (1.85-fold) when approximated by the elliptical sphere formula. Although few reported cases of uterine liposarcoma described the rate of increase in the tumor longitudinal axis or volume over time in postmenopausal patients with images, Kiuchi et al. [15] reported the only MRI finding of well-differentiated liposarcoma of the uterus that included the increase in mass over time after menopause. They found that the tumor size increased from 14.5 × 14 cm^2^ to 17 × 16 cm^2^ over six months, representing an increase of 14%–17%. On an annual basis, the tumor size of their patient increased by 30%, compared with 4% in our case. The increase in the size of uterine liposarcoma over time is estimated to vary considerably. In any case, if there is a slight difference in the interval between observations but the tumor size increases by 15% or more after menopause, liposarcoma should be suspected instead of lipoleiomyoma, and surgery should be planned. No other reports of uterine liposarcoma described an increase in tumor size over time.

If the lesion arises in the uterine body, then lipoma, lipoleiomyoma, and liposarcoma should be considered in the differential diagnosis based on the results of hematoxylin–eosin staining. Histologically, short spindle tumor cells were growing in coarse bundles in fibroadipose tissue. Disparities in the sizes of adipocytes and stromal cells were identified. Stromal cells also featured irregular nuclear findings and irregular and thickened nuclear margins. Because nuclear enrichment was observed, the lesion was considered a liposarcoma. As a subtype, a well-differentiated type was considered [17, 18].

From the results of immunohistology [21], p16 can be used for the differential diagnosis of liposarcoma [22]. In total, 83.3% of well-differentiated liposarcomas are positive for p16, whereas no deep-seated lipomas have been reported to express this protein. The finding partial positivity for p16 in this case affirmed a diagnosis of well-differentiated liposarcoma. In well-differentiated liposarcoma, it is useful to detect overexpression of these proteins by HDM2 and CDK4 gene amplification in the long arm (12q13–15) region of chromosome [12]. Immunostaining for MDM2 and CDK4 is positive in 60%–100% and 91% of well-differentiated liposarcomas, respectively. On the contrary, only 2%–4% of benign fatty tumors are positive for these proteins [23]. The findings of slight positivity for MDM2 and positivity for CDK4 in this case were in line with a minority of lesions for MDM2 and a majority of lesions for CDK4. Desmin, which was positive in this case, is derived from muscle cells. Uterine leiomyoma, lipoleiomyoma, and well-differentiated liposarcoma developmental processes were considered [11]. S100 protein is expressed by adipocytes but not atypical stromal cells. In this case, negativity for S100p indicated the presence of atypical stromal cells. Atypical stromal cells are positive for CD34. Partial positivity was noted for CD34 in this case, consistent with the majority of cases. Ki-67 is an index of growth markers indicating malignancy. A Ki-67 index higher than 10% indicates a high-grade tumor, whereas a value less than 10% indicates a low-grade tumor [24]. In this case, the Ki-67 index of 1% supported a diagnosis of well-differentiated liposarcoma.

Atypical lipoma-like tumor/well-differentiated liposarcoma is not a transformation (malignant transformation) from lipoma, spindle-shaped lipoma, or lipoleiomyoma, and the developmental mechanism is thought to be different. This is because extra circular chromosomes and giant marker chromosomes originating from the 12q14-15 region are frequently found in atypical lipoma-like tumors/well-differentiated liposarcoma. Most MDM2 and CDK4 gene amplifications are detected by FISH and immunostaining, but these gene amplifications are not observed in lipomas, lipoleiomyomas, spindle-shaped lipomas, etc. [19] However, McDonald et al. [11] stated that the vast majority of sarcomas are considered to arise de novo. Presenting photos of histopathological findings in which a liposarcoma part and a lipoleiomyoma or leiomyoma part exist adjacent to each other, our cases are the first to show an association of liposarcoma with lipoleiomyoma, which may be analogous to the rare occurrence of leiomyosarcomas arising in either leiomyoma or lipoleiomyoma.

Dedifferentiated liposarcoma is a highly malignant tumor, unlike well-differentiated liposarcoma. The morphology of the dedifferentiated component most often overlaps with that of undifferentiated pleomorphic sarcoma. Dedifferentiated areas exhibit a variable histological picture but most frequently resemble undifferentiated pleomorphic sarcoma or intermediate-high-grade myxofibrosarcoma [19]. Although dedifferentiation was originally defined by high-grade morphology, this case can be differentiated from dedifferentiated liposarcoma because mitoses are less than 2/HPF and the Ki67 index is 1%.

Since the 1977 report by Fujii et al. [1], 19 cases of uterine liposarcoma [1–16], including this report, have been confirmed (Table 1). To summarize the reported cases, uterine liposarcoma generally occurs during or after menopause. Excluding one case occurring in a 23-year-old woman [9], the age range of the patients was 45–78 years (mean, 60.5 years). The masses were usually larger than 9 cm (12/19 cases). They were detected on the basis of an abdominal mass or symptoms such as pain or bleeding. The reported symptoms included mass (tumor) sensation (*n* = 12), bleeding (*n* = 8), pain (*n* = 4), and mass pressure symptoms (*n* = 1). The extent of the lesion was often limited to the uterine body (corpus) (*n* = 12), and other sites included the cervix (*n* = 6), ligaments (*n* = 2), and uterine cavity (*n* = 1). Takeuchi et al. [7] was only (1/19 = 5.3%) able to achieve a preoperative correct diagnosis of liposarcoma from the excised specimen of the cervical mass. An initial treatment was most commonly TAH/BSO. Exceptional cases included the removal of a mass that spanned from the cervix to the vagina in an African patient [3] and the enucleation of a mass in the hope of preserving fertility [9] in the aforementioned 23-year-old patient. Patients with incomplete resection had a poor prognosis, and those in which the lesion was localized to the uterine body had a good prognosis. Meanwhile, lesions that arose in the cervix, intrauterine cavity, and sacral uterine ligament had a poor prognosis. Additionally, well-differentiated liposarcoma carried a good prognosis, whereas pleomorphic liposarcoma was associated with poor outcomes. No recurrence was detected in three patients with well-differentiated liposarcoma including our case. Meanwhile, four of the six patients with pleomorphic liposarcoma experienced recurrence. Patients who underwent complete removal of the lesion because of intrafibroid development of the uterine body had a good prognosis. In the five patients with round and myxoid liposarcoma, two patients with cervical and sacral uterine ligament development experienced recurrence, whereas the three patients with uterine body development and intrauterine fibroid development did not experience recurrence.

Several relevant articles were identified in the literature, but because the cited cases were similar, there was not much difference in the content [8, 9, 11–13, 15]. Summarizing the reported examples and examining their clinical treatment, it appears that complete tumor removal is planned for well-differentiated uterine liposarcoma, including removal of the uterus and bilateral adnexa. If the tumor cannot be completely removed, if it lacks uterine corpus development, or if it is not a well-differentiated lesion, then additional treatment such as anticancer drug treatment or radiation therapy is considered. There are three authors who chose chemotherapy as an additional treatment in the case of failure of complete resection or recurrence. Katabuchi et al. [4] chose a combination chemotherapy of cyclophosphamide, doxorubicin, and cisplatin at recurrence. Fadare and Khabele [12] chose a chemotherapy regimen of gemcitabine and docetaxel at recurrence. Schoolmeester et al. [14] opted for a chemotherapy regimen of gemcitabine and docetaxel at recurrence.

## Figures and Tables

**Table 1:** Liposarcoma of the uterus: review of literature.

Number	Authors	Year	Age (years)	GP	Symptom	Location	Tumor size	Operation	Histological subtype	Postoperative therapy	Recurrence	Treatment of recurrence	Next treatment	Follow-up
1	Fujii et al.	1977	51	P2	Bleeding	Corpus	7 × 7 × 6 cm	ATH+BSO	*Undifferentiated, nonmyxoid type*	RT				NED, 3 M (before 1 year)
2	Veliath et al.	1978	59	G4P4	Bleeding	*Cervix*	5 × 3 cm	ATH+BSO	Round cell		**Vaginal nodule, 1 M after operation**	**Excision**	**RT**	**N/A**
3	Obafunwa and Uguru	1990	45	P8	Bleeding	*Cervix*	Mass protruding from the cervix to the vagina	*The mass excised, the cervix marsupialised*	*Pleomorphic*	No	**Recurrence, 6 M**	**Conservative**	**Extensively excised**	**Not reported in hospital**
4	Katabuchi et al.	1990	54	G3P3	Tumor	*Left sacrouterine ligament*	8000 g	Resection of the tumor, ATH+BSO	Myxoid	No	**Recurrence, 4 M**	**Tumor resection, CT, RT, immunotherapy**	**Partial tumor resection, colostomy, uretero-**	**DOD, 2Y2M**
5	Schmidt and Doroszewski	1996	78	G2P2	Tumor	Corpus	13 cm	ATH+LtSO (previously Rt)	Myxoid and round cell	No				Free of disease, 3 Y
6	Schneebauer et al.	1996	67	N/A	Tumor	Corpus	15 × 13 cm	ATH+BSO	Myxoid	No				Free of disease, 3 Y
7	Takeuchi et al.	2000	49	Para 0	Bleeding	Cervix	4.0 × 3.0 × 1.0 cm	ATH+BSO+PLN	Well differentiated	No				Free of disease, 2 Y
8	Levine et al.	2003	62	G0P0	Pain	*Cervix, left sacrouterine ligament*	15 cm	ATH+BSO+PLN+PAN+O MT	*Pleomorphic*	No	**Pelvic recurrence, 9 M**	**Multiple pelvic nodules excised, RT**		**N/A**
9	Karateke et al.	2003	23	G0P0	Pain, tumor	*Cervix*	15 × 11 × 8 cm	*Extirpation of the mass, local resection of tumor could not be achieved completely, and minimal residual disease was left in place unfortunately*	Round and myxoid	CT				Free of disease. 7 M (before 1 year)
10	Hong et al.	2008	48	N/A	Pain, bleeding, tumor	Corpus	21 × 18 cm	ATH+BSO	Myxoid	No				Free of disease. 2 M (before 1 year)
11	McDonald et al.	2011	49	N/A	Tumor	Corpus	10.5 cm	ATH+BSO	*Pleomorphic*	No				Alive and well, 1 Y
12	McDonald et al.	2011	70	N/A	Tumor	Corpus	10 cm	ATH+BSO	Myxoid	No				Alive and well, 20 Y
13	McDonald et al.	2011	58	N/A	Bleeding, tumor	Corpus	18 cm	ATH+BSO	*Mixed pleomorphic and myxoid*	No				Alive and well, 2 Y
14	Fadare and Khabele	2011	62	N/A	Pain, tumor	Corpus	7 × 6.3 × 4.5 cm	ATH+BSO	*Pleomorphic*	RT	**Recurrence, 2 M**	**Anterior abdominal nodule was resected, CT**		**N/A**
15	Arai et al.	2014	77	3G3P	Tumor	Corpus	19 × 11 × 18 cm	ATH+BSO+OMT	Myxoid	No				No rec, 6 M (before 1 year)
16	Schoolmeester et al.	2016	70	N/A	Dysuria and urinary retention	Corpus and cervix	9.0 × 8.0 × 7.5 cm	MRH+BSO+PLN	*Pleomorphic*	No	**Numerous lung metastases, small and large intestine, a para-aortic lymph node**	**CT**		**DOD, 3 M**
17	Kiuchi et al.	2018	55	G6P3	Tumor, bleeding	Corpus	16 × 17 cm	ATH+BSO	Well differentiated	No				NED, 19 M
18	Lei et al.	2021	77	G4P4	Bleeding	*Uterine cavity*	6.0 × 5.0 × 1.7 cm	LH+BSO	*Dedifferentiated*	N/A				**N/A**
19	Hoshino et al.	2023	58	G4P2	Tumor	Corpus	4.8 cm	ATH+BSO	Well differentiated	No				NED, 10 M, under observation

G: gravidity; P: parity; N/A: not available, not applicable; MRH: modified radical hysterectomy; ATH: abdominal total hysterectomy; LH: laparoscopic hysterectomy; BSO: bilateral salpingo-oophorectomy; PLN: pelvic lymphadenectomy; PAN: para-aortic lymphadenectomy; OMT: omentectomy; meta: metastasis; RT: radiotherapy; CT: chemotherapy; NED: no evidence of disease; DOD: dead of disease. Italic texts: locations in recurrent or provable recurrent cases; incomplete operations; and undifferentiated, pleomorphic, or dedifferentiated in subtype. Bold texts: recurrent or provable recurrent cases.

## Additional Points

*Synopsis*. This study reports a case of uterine liposarcoma. Lipoleiomyoma that increases in size after menopause should be surgically removed and pathologically examined.
